# Language function following preterm birth: prediction using machine learning

**DOI:** 10.1038/s41390-021-01779-x

**Published:** 2021-10-11

**Authors:** Evdoxia Valavani, Manuel Blesa, Paola Galdi, Gemma Sullivan, Bethan Dean, Hilary Cruickshank, Magdalena Sitko-Rudnicka, Mark E. Bastin, Richard F. M. Chin, Donald J. MacIntyre, Sue Fletcher-Watson, James P. Boardman, Athanasios Tsanas

**Affiliations:** 1 Usher Institute, Medical School, University of Edinburgh, Edinburgh, UK; 2 MRC Centre for Reproductive Health, University of Edinburgh, Edinburgh, UK; 3 NHS Lothian-Neonatal Physiotherapy, Royal Infirmary of Edinburgh, Edinburgh, UK; 4 NHS Lothian-Neonatology, Royal Infirmary of Edinburgh, Edinburgh, UK; 5 Centre for Clinical Brain Sciences, University of Edinburgh, Edinburgh, UK; 6 Muir Maxwell Epilepsy Centre, Centre for Clinical Brain Sciences, University of Edinburgh, Edinburgh, UK; 7 Royal Hospital for Sick Children, Edinburgh, UK; 8 Division of Psychiatry, Deanery of Clinical Sciences, Royal Edinburgh Hospital, University of Edinburgh, Edinburgh, UK; 9 Salvesen Mindroom Research Centre, University of Edinburgh, Edinburgh, UK

## Abstract

**Background:**

Preterm birth can lead to impaired language development. This study aimed to predict language outcomes at 2 years corrected gestational age (CGA) for children born preterm.

**Methods:**

We analysed data from 89 preterm neonates (median GA 29 weeks) who underwent diffusion MRI (dMRI) at term-equivalent age and language assessment at 2 years CGA using the Bayley-III. Feature selection and a random forests classifier were used to differentiate typical versus delayed (Bayley-III language composite score <85) language development.

**Results:**

The model achieved balanced accuracy: 91%, sensitivity: 86%, and specificity: 96%. The probability of language delay at 2 years CGA is increased with: increasing values of peak width of skeletonized fractional anisotropy (PSFA), radial diffusivity (PSRD), and axial diffusivity (PSAD) derived from dMRI; among twins; and after an incomplete course of, or no exposure to, antenatal corticosteroids. Female sex and breastfeeding during the neonatal period reduced the risk of language delay.

**Conclusions:**

The combination of perinatal clinical information and MRI features leads to accurate prediction of preterm infants who are likely to develop language deficits in early childhood. This model could potentially enable stratification of preterm children at risk of language dysfunction who may benefit from targeted early interventions.

**Impact:**

- A combination of clinical perinatal factors and neonatal DTI measures of white matter microstructure leads to accurate prediction of language outcome at 2 years corrected gestational age following preterm birth.
- A model that comprises clinical and MRI features that has potential to be scalable across centres. It offers a basis for enhancing the power and generalizability of diagnostic and prognostic studies of neurodevelopmental disorders associated with language impairment.
- Early identification of infants who are at risk of language delay, facilitating targeted early interventions and support services, which could improve the quality of life for children born preterm.

## Introduction

An estimated 15 million infants are born preterm (before 37 weeks of gestation) annually worldwide.^1^ Although advances in neonatal intensive care have led to a decrease in infant mortality rates over time, survivors of preterm birth are at increased risk of long-term neurocognitive impairment.^2^ Preterm birth may lead to language deficits that persist into school age^3^ and are associated with a range of negative sequelae across the life span, including poor academic performance, poor social, emotional and behavioural functioning, and unemployment.^4,5^ Neurodevelopmental trajectories are amenable to early intervention, which presents a window of opportunity to have a profound, long-lasting effect on later life.^6^ Therefore, there is a clear unmet clinical need for early identification of those children who are at high risk of poor language development.

Multiple outcome studies have demonstrated associations between prenatal, neonatal, and postnatal factors and early neurodevelopmental outcomes for preterm infants.^7,8^ In addition, preterm birth is closely associated with generalized microstructural changes in cerebral white matter, inferred from diffusion tensor imaging (DTI) (fractional anisotropy [FA], mean, axial, and radial diffusivities [MD, AD, RD]), and alterations in these have been linked to language delay.^9^ However, it is rare for research to combine data from different modalities for the development of prediction models for neurodevelopmental outcomes.

Nonetheless, a few studies have built and validated tools for prediction of the composite outcome of neurodevelopmental impairment at 2 years corrected gestational age (CGA) for children born preterm. Tyson et al.^10^ investigated the clinical and demographic characteristics of a cohort of infants born before 26 weeks of gestation and found that the risk of adverse neurodevelopmental outcome at 18–22 months CGA was predicted using gestational age (GA), sex, exposure to antenatal corticosteroids, multiple birth, and birth weight. Ambalavanan et al.^11^ reported that neurodevelopmental impairment at 18–22 months CGA was predicted by combining sex, respiratory illness severity, and enlarged ventricular size, periventricular leukomalacia, or porencephalic cyst on cranial ultrasound. Vesoulis et al.^12^ developed a tool for prediction of risk of neurodevelopmental impairment at 18–24 months CGA. This tool comprised of ventilator days, mode of delivery, exposure to antenatal corticosteroids, retinopathy of prematurity (ROP) requiring surgery, and magnetic resonance imaging (MRI) findings (cerebellar haemorrhage size, cerebellar haemorrhage laterality, intraventricular haemorrhage grade, white matter injury).

However, deficits in different developmental domains require different therapies and targeted support strategies. Thus, tools for stratification of children at high risk of impairment in specific developmental domains would be valuable. Recently, Vassar et al.^13^ evaluated the predictive value of structural MRI and DTI variables for classification of very preterm infants at high versus low risk of language delay. They developed a model for prediction of language delay that included DTI variables in three brain regions and achieved 89% sensitivity and 86% specificity. Ball et al.^14^ revealed that distinct patterns of brain structure and microstructure following preterm birth are linked to specific clinical and environmental factors, and these patterns correlate with neurodevelopmental outcome at 18–24 months CGA. Language outcome was associated with specific neuroanatomic variation, which was linked to age at scan, need for continuous positive airway pressure, birth weight, GA at birth, parenteral nutrition, surfactant administration, and mechanical ventilation.

In view of this evidence, we hypothesized that a combination of clinical, environmental, and imaging factors derived from DTI that capture generalized white matter dysmaturation would potentially enhance the prediction of language outcomes at 2 years CGA following preterm birth. Blesa et al.^15^ demonstrated that histogram-based variables derived from DTI (peak width of skeletonized [PS] FA, MD, RD, and AD), which represent generalized water content and myelination, can be used as biomarkers of microstructural white matter alterations associated with preterm birth. The advantage of the histogram-based framework is that it is fully automated, captures generalized white matter dysmaturation that characterizes the encephalopathy of prematurity, is computationally inexpensive compared with tract-specific approaches, and has high inter-scanner reproducibility.^16^

A prediction tool that combines clinical data and imaging biomarkers for early language development is lacking, and yet timely identification of future language deficits has clinical and research implications, because it could stratify infants at most need for early interventions. Here we aimed to develop a machine learning model that accurately predicts typical versus delayed language outcomes at 2 years CGA using a parsimonious feature set derived from clinical, demographic, and histogram-based variables computed from neonatal brain DTI.

## Methods

### Participants

Participants were selected from a longitudinal cohort of preterm neonates born at ≤33 weeks of gestation at the Royal Infirmary of Edinburgh between February 2012 and August 2015.^17^ Selection from the larger cohort was based on availability of diffusion MRI (dMRI) scans at term-equivalent age and 2-year language outcome. Ethical approval was obtained from the UK National Research Ethics Service (NRES), South East Scotland Research Ethics Committee (NRES numbers 11/55/0061 and 13/SS/0143). Written informed consent from parents/carers was obtained for all neonates. Exclusion criteria for the study were congenital anomalies, chromosomal abnormalities, congenital infections or major overt parenchymal lesions (cystic periventricular leukomalacia, haemorrhagic parenchymal infarction), and post-haemorrhagic ventricular dilatation. Infants with a contraindication to MRI at 3 Tesla were also excluded.

### Clinical and demographic features

The selection of clinical and demographic features included in models was guided by extant literature linking biological and environmental exposures with neurocognitive development in preterm infants. Specifically, we studied the contribution towards prediction of language outcome at 2 years CGA of the following features: sex,^10,11,18,19^ GA (based on first trimester ultrasound),^10,18^ birth weight,^10,20^ maternal age,^21^ primiparity,^19^ twin status,^10,20^ maternal body mass index (BMI),^22^ medical history of maternal depression,^23^ administration of a complete course of antenatal corticosteroids for foetal lung maturation (defined as two doses 24 h apart), any antenatal corticosteroid exposure,^10,12,19,20^ administration of antenatal magnesium sulfate (MgSO_4_) for neuroprotection,^24^ mode of delivery (spontaneous vaginal delivery or caesarean section),^19^ total days requiring intubation while in the neonatal intensive care unit (NICU),^11,12,18^ bronchopulmonary dysplasia (defined as oxygen requirement at ≥36 weeks CGA),^19,20,25,26^ late-onset sepsis (defined as blood stream infection occurring ≥72 h postnatally with (a) bacterial pathogen isolated from blood culture or (b) blood culture growing coagulase-negative staphylococcus, along with one or more signs of generalized infection, and treatment with intravenous antibiotics for ≥5 days),^20^ necrotizing enterocolitis (NEC, defined as stages two or three according to the modified Bell’s staging for NEC^27^),^25,28^ ROP treated with laser therapy,^12,29^ and type of infant feeding at discharge from the neonatal unit (dichotomized as exclusive maternal breast milk versus exclusive formula or mixed feeding).^30^ All infants had placental histopathology performed and histological chorioamnionitis was defined using an established system.^31^ Maternal level of education (dichotomized as secondary school or below versus college, university or postgraduate studies)^18–20^ and socioeconomic status of the family, operationalized as Scottish Index of Multiple Deprivation 2016 (SIMD16) quintile, where 1 indicates the most deprived and 5 indicates the least deprived (https://www2.gov.scot/Topics/Statistics/SIMD), were also included.

### Image acquisition

Infants underwent a brain MRI scan at term-equivalent age (38–42 weeks GA) without sedation, during natural sleep after having been fed and swaddled. Vital signs were monitored throughout the scan, and hearing protection was provided for all neonates (MiniMuffs, Natus). All scans were supervised by a physician and a paediatric nurse trained in neonatal resuscitation.

A Siemens MAGNETOM Verio 3-Tesla MRI clinical scanner (Siemens Healthcare Gmbh, Erlangen, Germany) and 12-channel phased-array head coil were used to acquire dMRI data consisting of 11 T2-weighted and 64 diffusion-weighted (*b* = 750 s/mm^2^) single-shot, spin-echo, echo planar imaging volumes collected in the axial plane with 2 mm isotropic voxels (repetition time = 7300 ms, echo time = 06 ms, field of view = 256 mm, acquired matrix = 128 × 128, 50 contiguous interleaved slices with 2 mm thickness, acquisition time=9 min 29 s).

### Image analysis

**Fig. 1:**
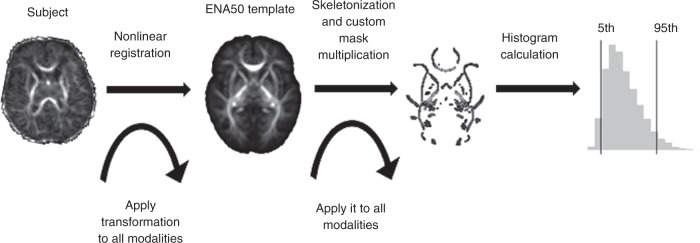
Scheme of the steps necessary for the calculation of the peak width of skeletonized DTI metrics. First, participants are registered to a template, then skeletonized and multiplied by a mask to calculate the histogram.

**Fig. 2:**
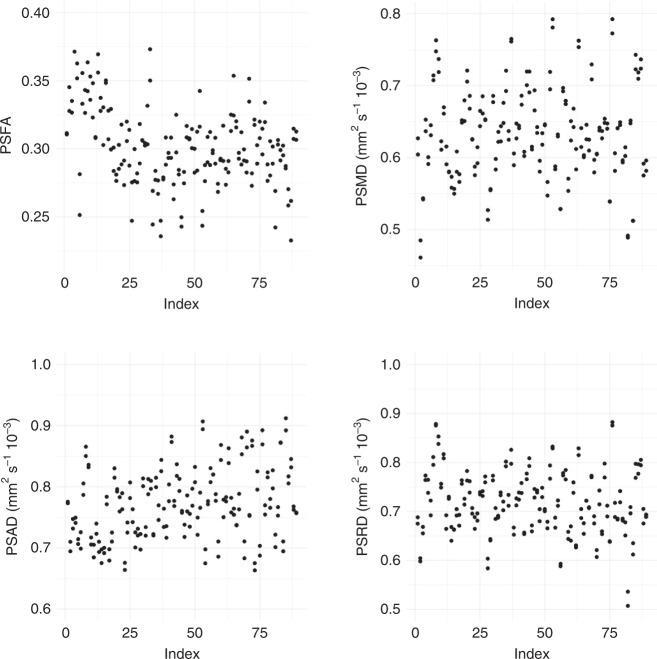
Scatterplots of the PSFA, PSMD, PSAD, and PSRD values for all participants. PSFA peak width of skeletonized fractional anisotropy, PSMD peak width of skeletonized mean diffusivity, PSAD peak width of skeletonized axial diffusivity, PSRD peak width of skeletonized radial diffusivity.

For each participant, the dMRI was denoised using a Marchenko-Pastur-PCA-based algorithm;^32,33^ eddy current and head movement were corrected using outlier replacement^34–36^ and bias field inhomogeneity correction was performed by calculating the bias field of the mean b0 volume and applying the correction to all the volumes.^37^ For each participant, PSFA, PSMD, PSRD, and PSAD were calculated using age-optimized methods described by Blesa et al.^15^ In summary, image data were registered to the Edinburgh Neonatal Atlas_50_^15^ using a tensor registration,^38^ and their DTI maps were calculated. Subsequently, the individual FA maps were projected into the template skeleton and multiplied by the atlas custom mask. Finally, the peak width of the histogram values within the skeletonized maps was calculated as the difference between the 95th and 5th percentiles.^16^ Figure 1 illustrates a summary of the process described. The code necessary to calculate histogram-based metrics can be found at https://git.ecdf.ed.ac.uk/jbrl/psmd. Figure 2 shows scatterplots of the values of the PS DTI metrics for all participants.

### Language outcome

All children took part in a developmental assessment with a trained clinician at 2 years CGA (median age 24.13, range 23.1–28.27 months) using the Bayley Scales of Infant and Toddler Development, Third Edition (Bayley-III).^39^ We used the Bayley-III language composite score (mean 100, SD 15) as the response variable. The clinical cut-off of 85 (i.e. 1 SD below the mean) was used in order to assign children into two distinct groups, thus creating a binary outcome; children whose score was <85 were considered to have moderate-to-severe language impairment, while scores ≥85 were considered as normal range or higher.^40^

### Data analysis

We compared three feature selection algorithms: (a) Boruta,^41^ (b) ReliefF expRank,^42,43^ and (c) random forests (RF) variable importance.^44^ The Boruta algorithm is a wrapper feature selection technique built around the RF learner, which uses *Z* score as the importance measure. In other words, it measures the importance of each feature by dividing the average loss of accuracy among all trees by the standard deviation of the accuracy loss. The basic idea of the ReliefF algorithm is to assign a ‘weight’ value to all features of a data set based on how well their values distinguish between the instances that are near to each other and thus how useful they are in predicting the response variable. The important features will have a large weight, while the redundant ones will have a low weight. In RF variable importance, variable importance is computed using the mean decrease in Gini index. We can measure the total amount that the Gini index is decreased by splits over a given feature, averaged over all trees. A large value indicates an important feature. In all cases, we obtain a feature ranking indicating in descending order their contribution towards prediction of the response variable. The final feature subset for each feature selection algorithm was selected using leave-one-out cross-validation (LOOCV), using only the training data set in each cross-validation iteration and following the process described by Tsanas et al.^45^ Subsequently, the selected feature subset was presented into a RF classifier^46^ in order to predict the binarized language composite score. Partial dependence plots (PDP)^47^ were constructed in order to assess how the selected features influence the prediction of the RF classifier. To quantify the strength of the association between the selected features, we used correlation analysis (the Spearman’s rank correlation coefficient was used to quantify the strength of the association between two continuous features, the phi coefficient was used to quantify the association between two binary features, and the point-biserial correlation coefficient was used to quantify the strength of the association between a continuous and a binary feature).

The data set is imbalanced since only 16% of the study group had a language composite score <85. To overcome the class imbalance problem in the data set, we explored different data balancing techniques: under-sampling of the majority class, over-sampling of the minority class, and the synthetic minority over-sampling technique (SMOTE),^48^ which has been previously used in similar unbalanced applications in the healthcare domain.^49–53^ We found that SMOTE yields the best results, which are presented in the paper. SMOTE is a training data enrichment method, where the minority class is over-sampled by creating new synthetic samples, to create a balanced data set. For each minority class sample, the *k* minority class nearest neighbours were identified (using the suggestion of Chawla et al. with *k* = 5) and synthetic samples were introduced along the line segments joining any or all of the *k* minority class nearest neighbours. Model validation was implemented using LOOCV. LOOCV involves holding out a single observation to be used as the test set, while the learner is trained using the remaining *n* − 1 observations (*n* is the total number of observations). The process is repeated *n* times and each time a different observation from the original data set is used as the test set. The result is *n* estimates of the test error. The final test error rate is the average of these *n* test error estimates. The accuracy of the model was assessed by constructing a confusion matrix, which is a contingency table of the observed and predicted classes. Missing data for both numeric and categorical features were imputed using multiple imputation by chained equations (five imputed data sets were created in each LOOCV iteration),^54,55^ based only on the information in the training set independently within each LOOCV iteration. Data analysis was conducted in R. The R packages used were: tidyverse, dplyr, caret, randomForest, CORElearn, Boruta, mice, ggplot2, DMwR, Hmisc, RGraphics, grid, gridExtra, and gridGraphics.

## Results

Two-year language data and dMRI of the brain at term-equivalent age were available from 89 children; demographic and clinical characteristics of the study population are presented in Table 1. At median age 24.13 months (range 23.24–28.27 months), 14 children had a language composite score <85. The percentage of missing values in the data set was 0.2% (1 participant had missing histological chorioamnionitis data, 2 participants had missing SIMD16, and 3 participants had missing maternal BMI).

**Table 1:** Demographic and clinical characteristics of the study group.

Characteristics	Neonates with language composite score ≥85 (*N* = 75)	Neonates with language composite score <85 (*N* = 14)
*Antenatal*		
Any antenatal corticosteroids	73 (97)	11 (79)
Complete course of antenatal corticosteroids	56 (75)	5 (36)
Antenatal MgSO_4_ for foetal neuroprotection	39 (52)	8 (57)
*Perinatal*		
Sex		
Male	35 (47)	12 (86)
Female	40 (53)	2 (14)
GA (weeks)	28.84 ± 3.28 (23.28 to 33)	28.92 ± 2.18 (23.28 to 30.28)
Birth weight (g)	1137 ± 376.5 (568 to 1500)	1040 ± 410 (550 to 1635)
Birth weight *z* score	−0.16 ± 1.15 (−2.63 to −1.17)	0.12 ± 1.30 (−1.77 to −1.0)
Apgar score at 5 min	7.5 ± 2 (2 to 9)	8 ± 2 (5 to 9)
Mode of delivery		
SVD	32 (43)	3 (21)
Caesarean section	43 (57)	11 (79)
Primiparity	52 (69)	8 (57)
Twin status	21 (28)	10 (71)
*Postnatal*		
BPD	25 (33)	6 (43)
LOS	20 (27)	5 (36)
NEC	5 (7)	0 (0)
ROP	5 (7)	1 (7)
Histologic chorioamnionitis	22 (31)	3 (21)
Days of intubation	1 ± 5.5 (0 to 39)	1 ± 1 (0 to 43)
Feeding at discharge		
Exclusive maternal breast milk	36 (48)	2 (14)
Exclusive formula or mixed feeding	39 (52)	12 (86)
*Demographics*		
Maternal race		
Asian	5 (6)	0 (0)
White	66 (88)	13 (93)
White/Asian	1 (1)	0 (0)
White/Black	2 (2)	1 (7)
Other mixed	1 (1)	0 (0)
Maternal age (years)	32 ± 8 (17 to 43)	33 ± 8 (23 to 40)
Maternal BMI	24.7 ± 4.5 (17.4 to 43)	24.1 ± 6.9 (18 to 30.9)
Medical history of maternal depression	10 (13)	1 (7)
Maternal education		
Secondary school or below	33 (44)	6 (43)
College/University/postgraduate studies	42 (56)	8 (57)
SIMD16 quintile		
1	8 (11)	3 (21)
2	23 (32)	3 (21)
3	11 (15)	3 (21)
4	12 (16)	3 (21)
5	19 (26)	2 (14)
*Histogram-based variables derived from DTI*		
PSFA	0.3 ± 0.03 (0.25 to 0.37)	0.32 ± 0.02 (0.24 to 0.36)
PSMD	0.63 ± 0.07 (0.49 to 0.79)	0.61 ± 0.07 (0.49 to 0.79)
PSRD	0.71 ± 0.09 (0.54 to 0.88)	0.72 ± 0.07 (0.6 to 0.83)
PSAD	0.77 ± 0.08 (0.68 to 0.89)	0.76 ± 0.09 (0.69 to 0.91)
*Bayley-III*		
Language composite score	100 ± 24 (86 to 132)	77 ± 11 (56 to 83)

Variables are presented in the form median ± IQR (range) or number (%).

**Fig. 3:**
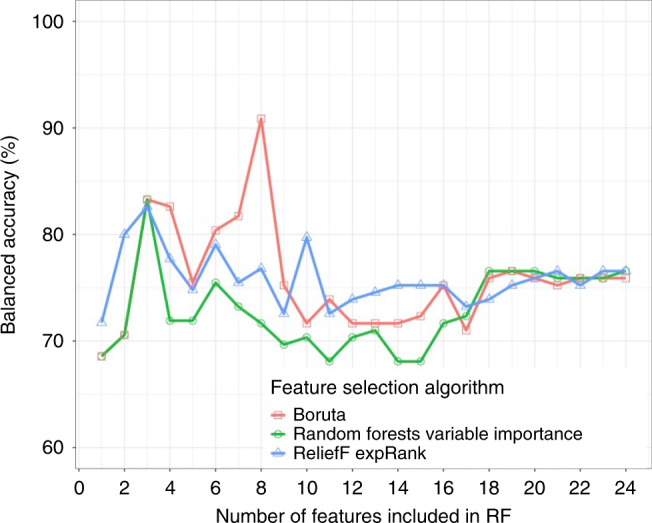
Comparison of out-of-sample LOOCV balanced accuracy results of the random forests classifier using the features selected by each of the three feature selection algorithms.

**Fig. 4:**
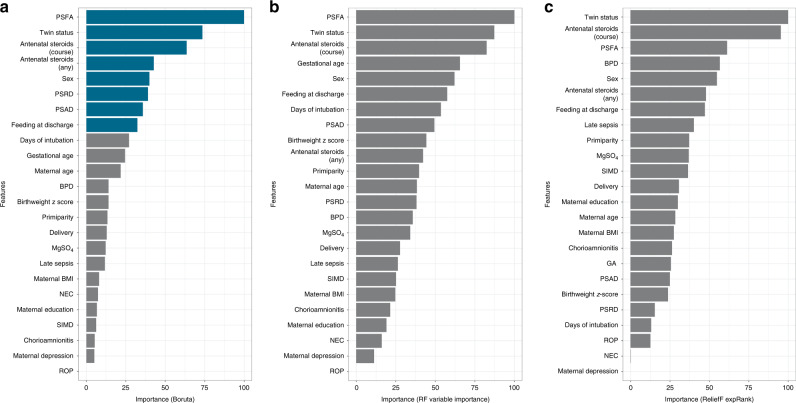
Feature importance plots. **a** Importance attributed to each feature by the Boruta algorithm. The first eight features coloured in blue (PSFA, twin status, course of antenatal steroids, any antenatal steroids, sex, PSRD, PSAD, feeding at discharge) are the jointly most predictive features towards the prediction of language outcome. **b** Importance attributed to features by RF variable importance. **c** Importance attributed to features by ReliefF expRank. Computation of feature importance depends on the feature selection algorithm used and is expressed relative to the maximum.

**Fig. 5:**
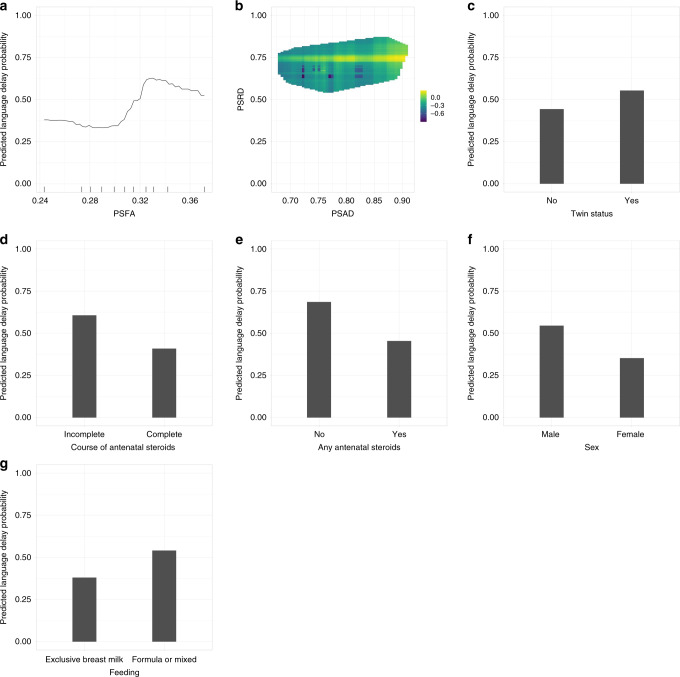
Partial dependence plots for the eight features selected by Boruta and used in the random forests classifier. **a** The predicted language impairment probability rises with increasing PSFA values. **b** 3D plot of PSRD and PSAD. The predicted language impairment probability rises with increasing PSRD and PSAD values. **c** A twin pregnancy increases the predicted probability of language impairment. **d** An incomplete course of antenatal corticosteroids increases the predicted probability of language impairment. **e** No exposure to any antenatal steroids increases the predicted probability of language impairment. **f** Female sex reduces the predicted probability of language impairment. **g** Feeding with exclusive breast milk reduce the predicted probability of language impairment. Language composite score <85 at 2 years CGA is more likely following a twin pregnancy, an incomplete course of antenatal corticosteroids, or no exposure to any antenatal steroids. Female sex and feeding with exclusive breast milk reduce the risk of future language delay.

**Fig. 6:**
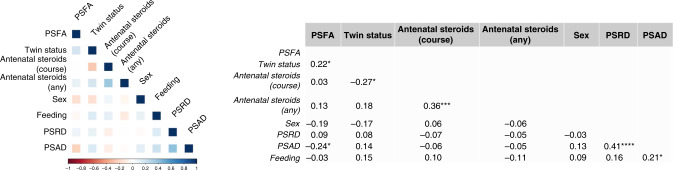
Correlogram and correlation matrix of the eight most important features selected by the Boruta algorithm. *p < 0.05, ***p < 0.001, ****p < 0.0001.

Figure 3 illustrates the out-of-sample performance of the RF classifier (trained on approximately 150 samples in each LOOCV iteration) as a function of the number of features selected by the different feature selection algorithms. These data show that feeding a subset of eight features selected by the Boruta feature selection algorithm (a wrapper feature selection technique built around the RF learner) to the RF classifier gives the highest balanced accuracy. The selected feature subset comprises PSFA, twin status (yes or no), antenatal steroid exposure (complete or incomplete course), any antenatal steroid exposure (yes or no), sex (male or female), PSRD, PSAD, and feeding at discharge from the NICU (exclusive maternal breast milk versus exclusive formula or mixed feeding). Figure 4 shows the importance attributed to each feature by each of the feature selection algorithms. PSFA, twin status, the course of antenatal steroid exposure, any antenatal steroid exposure, sex, PSRD, PSAD, and feeding are the jointly most predictive features towards the prediction of the binarized language outcome. PDP were used to visualize relationships between the selected features and the response based on our model (see Fig. 5). The PDP provide insight into the effect of changing one or two features in terms of the model’s prediction (binary response variable, indicating whether language composite score <85). Regarding the histogram-based variables derived from DTI, the PDP show that the predicted language impairment probability rises with increasing PSFA, PSRD, and PSAD values. PSRD and PSAD are presented in the same plot because they are highly correlated as illustrated in the correlogram and correlation matrix in Fig. 6. Language composite score <85 at 2 years CGA is more likely following a twin pregnancy, an incomplete course of antenatal corticosteroids, or no exposure to antenatal steroids. Female sex and feeding with exclusive breast milk reduce the risk of future language delay.

Table 2 shows the confusion matrix of the out-of-sample classification performance of the RF classifier when mapping the selected feature subset (i.e., PSFA, twin status, antenatal corticosteroid exposure, sex, PSRD, PSAD, and feeding at discharge) to the binarized language composite score. Our model achieved balanced accuracy: 91%, sensitivity: 86%, and specificity: 96%.

**Table 2:** Confusion matrix summarizing the out-of-sample findings using LOOCV.

Prediction	Reference	
	Language composite score <85	Language composite score ≥85
Language composite score <85	12 (14%)	3 (3%)
Language composite score ≥85	2 (2%)	72 (81%)

Finally, we repeated the analysis to investigate separately the performance of the model when presented only with either clinical or MRI features, which led to reduced model performance. As shown in Table 3, the model that comprises clinical and MRI features outperformed the models using only clinical or MRI features. The combination of clinical and DTI features enhances the prediction of language outcomes at 2 years CGA following preterm birth.

**Table 3:** Model performance using (a) only clinical features, (b) only MRI features, and (c) the combination of clinical and MRI features.

Models	Balanced accuracy	Sensitivity	Specificity
Clinical features	83%	79%	87%
MRI features	81%	86%	76%
Clinical and DTI features	91%	86%	96%

## Discussion

We developed a parsimonious machine learning model that accurately identifies preterm infants who are likely to develop language impairment in early childhood. We explored the predictive value of 24 clinical, demographic, and brain imaging features and found that a robust subset of eight clinical characteristics and imaging biomarkers best predicts a language composite score <85 on the Bayley-III: PSFA, PSRD, PSAD, twin status, administration of an incomplete course of antenatal corticosteroids, no exposure to antenatal corticosteroids, male sex, and feeding with exclusive formula milk or mixed formula and breast milk. Overall, we demonstrated out-of-sample balanced accuracy: 91%, sensitivity: 86%, and specificity: 96%.

Feature selection was conducted by comparing three feature selection algorithms: (a) Boruta, (b) ReliefF expRank, and (c) RF variable importance. Feature selection methods can be broadly considered into three main categories: filter, wrapper, embedded methods. Filter feature selection methods work independently of a statistical learner relying on the general statistical properties of the data and thus select a feature subset that is not tuned or optimized towards a specific learning algorithm. Wrapper methods take a particular machine learning method into account in order to choose the best subset of the original features. They evaluate multiple models by training and testing in the feature space, thus optimizing the performance of the particular machine learning model that was used. Embedded methods choose the subset of features while the learning model is being constructed. This means that the resulting feature subset is specific to a particular learning algorithm. We chose to use a feature selection algorithm from each main category for our exploration; ReliefF is a filter technique, Boruta is wrapper feature selection technique built around the RF learner, and RF variable importance is an embedded method. The use of ReliefF and the RF importance have been extensively used and validated in many different applications and we have previously conducted a thorough empirical study^56^ where they performed very competitively against many established feature selection approaches. In general, we would expect a wrapper or embedded method to perform better for a particular choice of a classifier, although it might not necessarily generalize very well with the choice of different classifiers.

Our findings suggest that PSFA, PSRD, and PSAD, which detect generalized white matter microstructural alterations in preterm infants compared to infants born at term,^15^ are predictive of impaired language development at 2 years CGA. We explored the predictive value of whole-brain measures of PS DTI metrics, instead of tract-specific segmentations, because preterm brain dysmaturation is a substantially generalized process,^57^ and language development draws on broad cognitive capacities. We have found that the probability of language delay is higher with increased PSFA, PSRD, and PSAD. These features are consistent with delayed myelination, less coherent white matter organization, and altered axonal integrity in the preterm brain.^15,58^ Previous research has also shown that abnormalities in brain structure following preterm birth are correlated with long-term neurodevelopmental outcome.^59^

The data show that twin status is associated with increased risk of impaired language development. This finding is consistent with studies in the extant literature which have found that multiple pregnancy is associated with neurodevelopmental impairment^10,20,60^ and language delay^61^ at 2 years CGA. Language delay in twins can be attributed to postnatal environmental factors;^62,63^ twins receive a less focussed and less elaborated communicative interchange with their parents than do singletons. Thorpe et al.^62^ compared families with twins to families with pairs of closely spaced singletons. This study found that language delay in twins compared to singletons may be explained by patterns of parent–child interaction and communication. Antenatal corticosteroid administration is associated with lower risk of language deficits, which has been previously proved by research.^10,12^ Our findings suggest that male sex is a risk factor for language impairment in early childhood, consistent with previous studies that have associated male sex with poorer neurodevelopmental outcome following preterm birth.^10,11,18,19^

Moreover, previous work has shown that exclusive breast milk feeding in the weeks following preterm birth can enhance brain development,^30^ and in the general population breast milk intake in infancy is associated with improved performance on intelligence tests.^64^ In line with this, we found that exclusive breastfeeding is associated with improved language outcomes compared to formula feeding or mixed breast and formula feeding. It is surprising that GA at birth was not included in the final feature set. However, its influence on long-term outcome may be captured by PSRD and PSAD, which are strongly correlated with GA at birth.^15^

This study is the first to investigate the use of PS DTI metrics as predictors for language development in the preterm population. The advantage of using these image biomarkers is that their calculation is fully automated, computationally inexpensive, and has high inter-scanner reproducibility,^16^ meaning that they can be easily obtained for preterm neonates who undergo a dMRI scan at term-equivalent age and can be used for multi-centre studies. Thus, our model comprises features that can be easily obtained for future clinical application.

Hitherto, few studies have focussed on developing and validating prediction models for early neurodevelopmental outcomes for children born preterm. Most tools predict the composite outcome of neurodevelopmental impairment.^10–12^ However, deficits in different developmental domains require different interventions. Therefore, tools for timely identification of children at risk of impairment in specific developmental domains are valuable. The developed model predicts language deficits at 2 years CGA. Recently, a model was developed for classification of very preterm infants at high versus low risk for language delay, which achieved 89% sensitivity and 86% specificity.^13^ That model included DTI variables in three brain regions: MD of right sagittal stratum and right inferior occipital gyrus and AD of right lingual gyrus. However, whole-brain calculation of DTI variables is computationally expensive; hence, we investigated the predictive value of histogram-based variables derived from DTI. We have shown that combining DTI metrics with perinatal factors, along with the use of advanced machine learning techniques, can further improve identification of children at risk of language impairment.

The main strength of our study is that we had a longitudinal cohort of preterm infants that is deeply phenotyped with brain imaging and biological information that enabled us to investigate a large number of clinical, demographic, social, and DTI variables. We acknowledge some limitations in our study. The sample size is relatively small, and this is a single-centre study, so despite our best efforts with standard model validation techniques to assess model generalization we would need to further validate findings in a different cohort. Nonetheless, the study population was fairly representative of NICU populations in terms of comorbidities that have been associated with long-term neurodevelopmental outcomes. In addition, cortical grey matter was not assessed in this study. We focussed on alterations in white matter microstructure, since it is the most consistently abnormal finding in preterm infants, by measuring a functionally tractable property using a tool that is readily applied to clinical image data. Future studies could aim to validate our model in additional external cohorts and also apply machine learning techniques for prediction of motor, cognitive, and social–emotional outcomes for children born preterm.

## Conclusion

A combination of clinical perinatal factors and neonatal DTI measures of white matter microstructure best predict language impairment at 2 years after preterm birth. This model has the potential to enable clinicians identify infants who are at risk of language delay, thus facilitating targeted early intervention and support services. The model comprises clinical and MRI features that have potential to be scalable across centres, so it offers a basis for enhancing the power and generalizability of diagnostic and prognostic studies of neurodevelopmental disorders associated with language impairment.

## Supplementary information

Supplementary abbreviations
