# Supplementary material for: Language function following preterm birth: prediction using machine learning
Source: Pediatr Res. 2021 Oct 11;92(2):480–9. doi: 10.1038/s41390-021-01779-x (PMC8503721; doi:10.1038/s41390-021-01779-x)
Supplement: Supplementary file 1 — Supplementary abbreviations [file 41390_2021_1779_MOESM1_ESM.docx]

| AD | Axial diffusivity |
| --- | --- |
| Bayley-III | Bayley Scales of Infant and Toddler Development, Third Edition |
| BMI | Body mass index |
| BPD | Bronchopulmonary dysplasia |
| CGA | Corrected gestational age |
| dMRI | Diffusion MRI |
| DTI | Diffusion tensor imaging |
| ENA_50_ | Edinburgh Neonatal Atlas_50_ |
| FA | Fractional anisotropy |
| GA | Gestational age |
| LOOCV | Leave-one-out cross-validation |
| LOS | Late onset sepsis |
| MD | Mean diffusivity |
| MgSO_4_ | Magnesium sulphate |
| MRI | Magnetic resonance imaging |
| NEC | Necrotizing enterocolitis |
| NICU | Neonatal intensive care unit |
| NRES | National Research Ethics Service |
| PDP | Partial dependence plot |
| PSAD | Peak width of skeletonized axial diffusivity |
| PSFA | Peak width of skeletonized fractional anisotropy |
| PSMD | Peak width of skeletonized mean diffusivity |
| PSRD | Peak width of skeletonized radial diffusivity |
| RD | Radial diffusivity |
| RF | Random forests |
| ROP | Retinopathy of prematurity |
| SIMD16 | Scottish Index of Multiple Deprivation 2016 |
| SMOTE | Synthetic minority over-sampling technique |
| SVD | Spontaneous vaginal delivery |
